# Sensory evoked gamma oscillation reduces white matter loss and preserves myelin in Alzheimer's disease: neuroimaging and proteomics findings

**DOI:** 10.1002/alz70856_097702

**Published:** 2025-12-24

**Authors:** Mihaly Hajos

**Affiliations:** ^1^ Yale University School of Medicine, New Haven, CT, USA; Cognito Therapeutics, Cambridge, MA, USA

## Abstract

**Background:**

Neuroimaging findings have shown that white matter volume loss and demyelination critically contribute to brain atrophy in Alzheimer's disease (AD). Notably, demyelination occurs in the presymptomatic AD stages, preceding the formation of amyloid‐β plaques and neurofibrillary tangles. Recently, it has been reported that sensory evoked steady‐state gamma oscillation attenuates cognitive and functional decline and brain volume loss in AD patients (Hajos et al., 2024). Given the established role of neuronal network oscillation in oligodendrocyte activity promoting myelination (Hant et al., 2016), it was hypothesized that sensory evoked gamma oscillation could preserve myelin in in AD. This study evaluated this hypothesis using neuroimaging and CSF proteomics results from clinical trials participants with AD.

**Method:**

Neuroimaging data were collected from participants with AD who received daily, 40Hz sensory stimulation (Spectris^TM^ investigational medical device) for six months, while sham participants received sham stimulation (OVERTURE; NCT03555280). MRI data were collected at baseline, 3‐ and 6‐months. MRI T1 images and T1w/T2w ratios were used to estimate white matter (*n* = 38) and corpus callosum (*n* = 50) volumes and myelination (*n* = 36), respectively. Unbiased proteomics analysis was done from CSF samples from participants (*n* = 10) with amyloid positive mild cognitive impairment (FLICKER; NCT03543878) who received daily treatment with Spectris for 8 weeks. CSF samples were collected at baseline, 4 amd 8 weeks of treatment.

**Result:**

Sensory evoked gamma oscillation significantly reduced total and regional white matter atrophy and myelin content loss in active treatment compared to sham treatment participants. Most significant differences were observed in the entorhinal region and corpus callosum. Treatment altered CSF proteins linked to AD‐related brain proteome modules, including those involved in myelination, synaptic and neuroimmune functions, and regulation of cellular lipid transportation. Biological pathway analysis revealed that most affected pathways were associated with lipoproteins, cholesterol, phospholipids processing and phosphatidylcholine biosynthesis.

**Conclusion:**

Current results indicate that sensory evoked steady‐state gamma oscillation reduces white matter atrophy and preserves myelin content. Although proteomics results suggest pleiotropic effects on multiple AD pathways, significant impacts were observed on proteins and biological processes linked to myelination. Potential clinical benefits of Spectris treatment may be related to treatment effects on white matter and myelin.

